# Bilateral Minithoracotomy Approach for Off-Pump Coronary Artery Bypass Grafting in a Patient With Retrosternal Gastric Tube Reconstruction

**DOI:** 10.1016/j.atssr.2026.03.008

**Published:** 2026-03-26

**Authors:** Shinichi Tsumaru, Yuki Wada, Akira Marui, Nobuhisa Ohno

**Affiliations:** 1 Department of Cardiovascular Surgery, Kokura Memorial Hospital, Kitakyushu, Japan

## Abstract

Retrosternal gastric tube reconstruction after esophagectomy can make coronary artery bypass grafting technically challenging because the gastric tube often occupies the retrosternal space and may lie close to major mediastinal structures. We report a case of multivessel coronary artery disease in a patient with previous esophagectomy and retrosternal gastric tube reconstruction. To avoid gastric tube injury and enable safe multivessel revascularization, off-pump coronary artery bypass grafting was performed through bilateral minithoracotomies, with proximal anastomosis through a right minithoracotomy and distal anastomoses through a left minithoracotomy.

Retrosternal gastric tube reconstruction is now a standard reconstructive procedure for esophagectomy performed for esophageal cancer.^1^ However, this altered mediastinal anatomy can make coronary artery bypass grafting (CABG) challenging because the gastric tube often occupies the retrosternal space and may lie close to the internal mammary arteries (IMAs) or ascending aorta, thus rendering median sternotomy unsafe. Minimally invasive CABG offers an alternative, although reports of multivessel revascularization remain limited.^2^^,^^3^ We describe successful multivessel off-pump CABG through bilateral minithoracotomies in this challenging anatomic context.

A 66-year-old man underwent coronary angiography during evaluation of inferior wall asynergy, which revealed multivessel coronary artery disease involving the left main trunk. The angiogram demonstrated 75% stenosis of the left main trunk, 90% stenosis of the proximal left anterior descending artery (#6-7), 75% stenosis of the obtuse marginal branch (#11), and chronic total occlusion of the proximal right coronary artery (#1) with collateral flow from the left circumflex artery (Figure 1).

**Figure 1:**
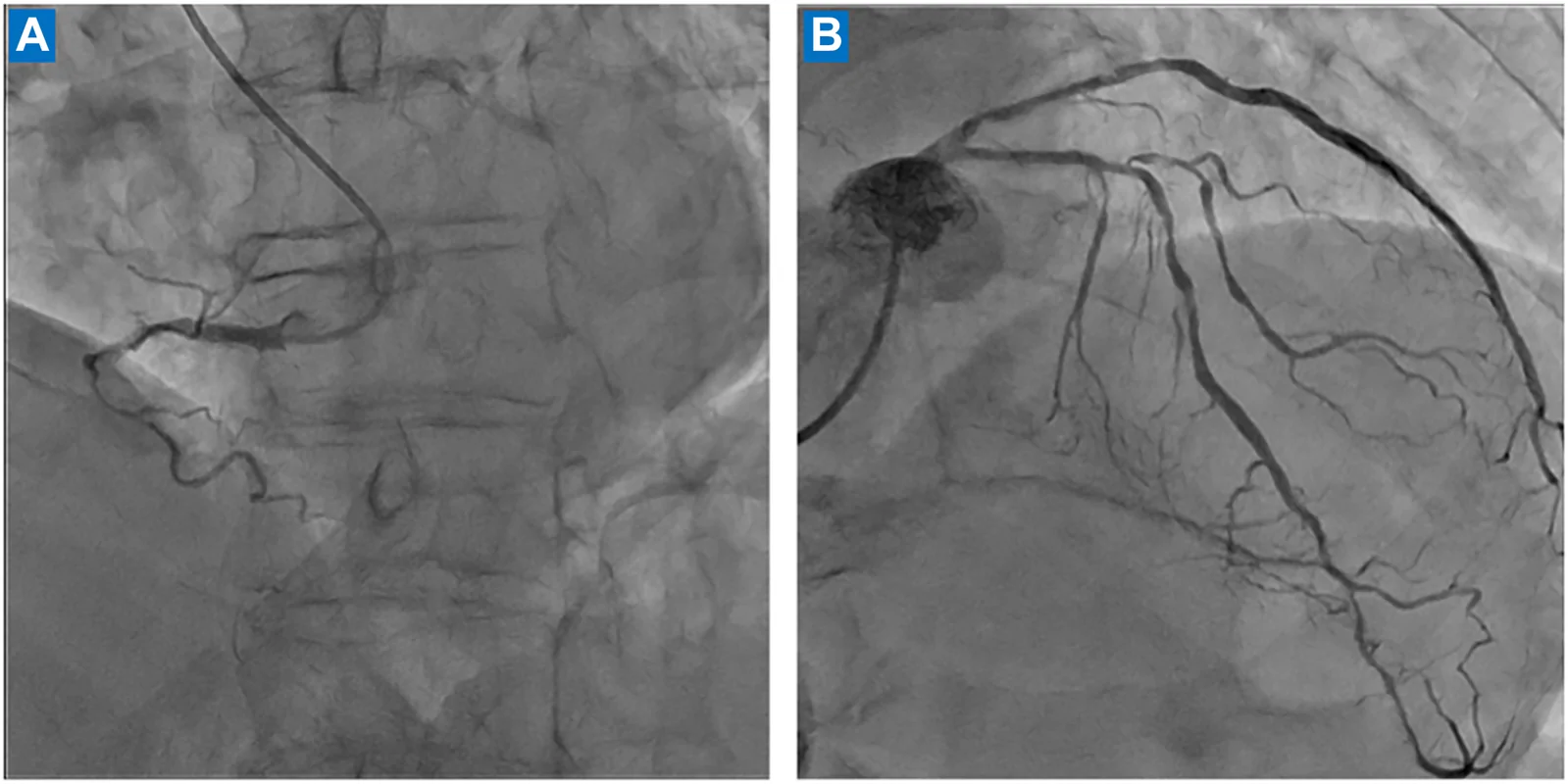
(A, B) Coronary angiography revealed 3-vessel coronary artery disease involving the left main trunk.

He had undergone esophagectomy with retrosternal gastric tube reconstruction for esophageal cancer at the age of 65 years. His medical history included type 2 diabetes mellitus and a significant smoking history. Given the complexity of multivessel disease and the presence of left main trunk involvement, percutaneous coronary intervention was deemed unsuitable, and CABG was indicated.

Preoperatively, we performed contrast-enhanced computed tomography (CT) to evaluate the reconstructed gastric tube, the IMA, and the ascending aorta. The gastric tube was markedly dilated and was located directly behind the sternum, with the left IMA lying in close contact with it (Figures 2A-2D). The aortic wall was deemed suitable for proximal anastomosis but was positioned slightly rightward in the mediastinum (Figures 2A, 2C). We considered that a median sternotomy would carry a high risk of gastric tube injury and selected a left minithoracotomy to access the coronary arteries. Harvesting the left IMA was also considered unsafe because of its close proximity to the dilated gastric tube, and a saphenous vein graft (SVG) was planned for revascularization. However, because the ascending aorta—the intended site for proximal anastomosis—was positioned slightly rightward in the mediastinum while the gastric tube occupied the left retrosternal space, we considered proximal anastomosis through the left minithoracotomy to be difficult. Consequently, a right minithoracotomy was planned for the proximal anastomosis, and a left minithoracotomy was planned for the distal anastomosis.

**Figure 2:**
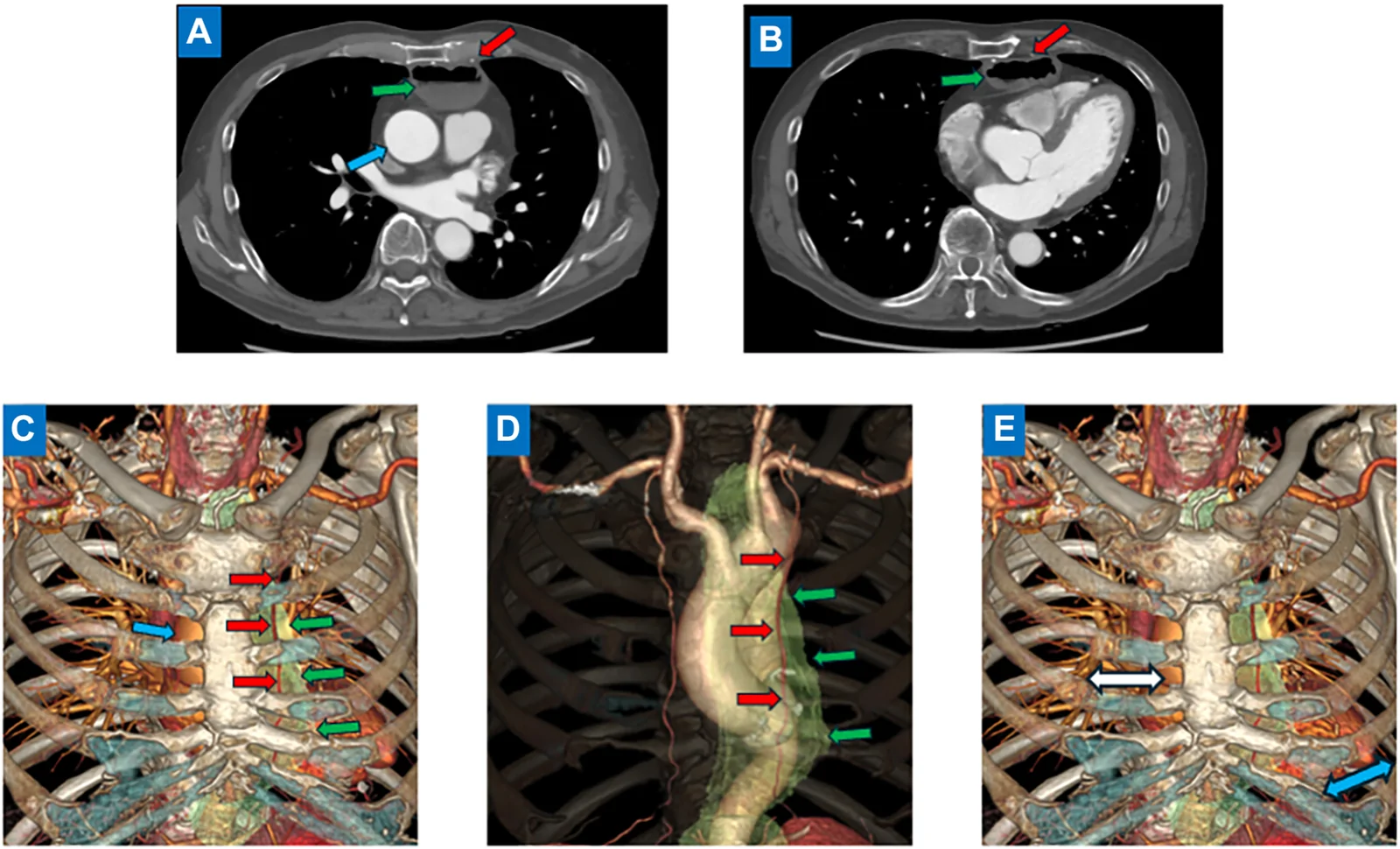
Preoperative computed tomography. (A) Axial image at the second intercostal level. A markedly dilated gastric tube lies directly behind the sternum (green arrow) in close contact with the left internal mammary artery (red arrow), and the ascending aorta (blue arrow) is positioned slightly rightward relative to the gastric tube. (B) Axial image at the fifth intercostal level. A markedly dilated gastric tube lies directly behind the sternum (green arrow) in close contact with the left internal mammary artery (red arrow). (C) A 3-dimensional computed tomographic reconstruction with the rib cage. The spatial relationships among the gastric tube, the left internal mammary artery, and the rightward-shifted ascending aorta correspond to those shown in (A, arrows). (D) A 3-dimensional computed tomographic reconstruction without the rib cage demonstrates the same anatomic configuration as (A, arrows). (E) A 3-dimensional computed tomographic reconstruction with the rib cage. The right minithoracotomy at the third intercostal space (white double-headed arrow) and the left minithoracotomy at the fifth intercostal space (blue double-headed arrow) are indicated.

General anesthesia was induced, and the patient was placed in a right semilateral decubitus position. A right minithoracotomy was performed in the third intercostal space to expose the ascending aorta (Figure 2E). The aortic wall appeared suitable for proximal anastomosis. A left minithoracotomy was then performed through the fifth intercostal space to identify the distal target vessels (Figure 2E). Two SVGs had been harvested from both lower extremities. After systemic heparinization, proximal anastomosis was performed through the right minithoracotomy. Using the Heartstring III Proximal Seal System (Getinge), an aortic punch was created, and the first SVG was anastomosed to the ascending aorta. The second SVG was then anastomosed to the first SVG by using the piggyback technique. The 2 grafts were subsequently guided into the left thoracic cavity for distal anastomoses. The second SVG was sequentially anastomosed to the obtuse marginal branch (#12) and the posterior descending artery (#4PD), whereas the first SVG was anastomosed to the distal left anterior descending artery (#8). All graft flows were satisfactory. The patient’s postoperative course was uneventful, and postoperative computed tomography confirmed graft patency (Figure 3).

**Figure 3:**
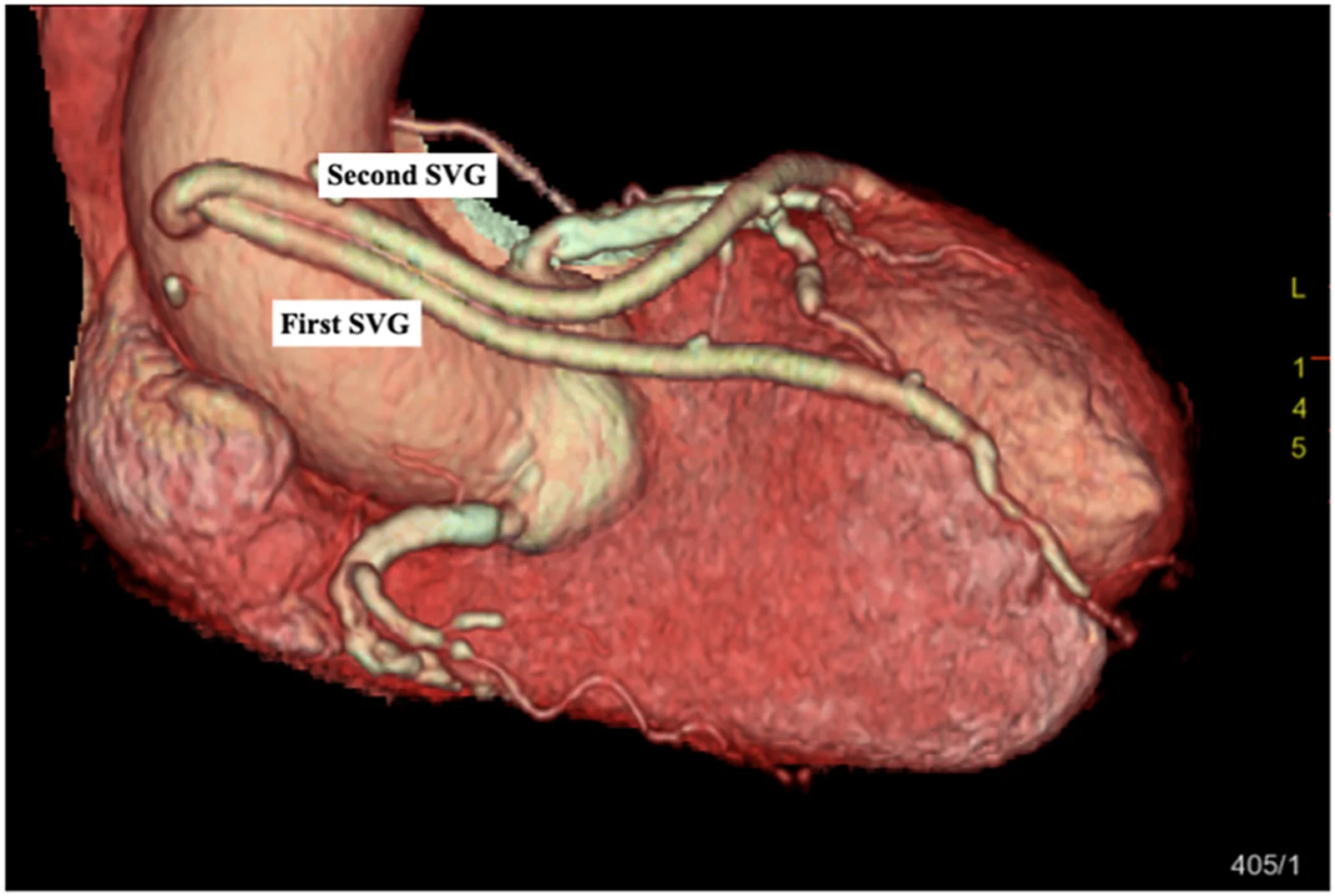
Postoperative contrast-enhanced computed tomography demonstrated patency of all bypass grafts. SVG = saphenous vein graft.

## Comment

We present a case of complete revascularization performed through bilateral minithoracotomies in a minimally invasive CABG approach in a patient who had previously undergone esophagectomy with retrosternal gastric tube reconstruction for esophageal cancer. This strategy allowed us to avoid injury to the reconstructed gastric tube while achieving 3-territory revascularization.

Patients with a history of esophagectomy and retrosternal gastric tube reconstruction present technical challenges for CABG. The gastric tube frequently occupies the retrosternal space, thus making median sternotomy unsafe because of the possibility of gastric tube injury or postoperative mediastinitis. Previous reports have described various approaches, including left thoracotomy, right thoracotomy, and carefully planned sternotomy, but each carries limitations depending on the position of the gastric tube, the degree of adhesions, the target coronary anatomy, and the position of the ascending aorta for proximal anastomosis.^1^, ^2^, ^3^, ^4^, ^5^ Harvesting the IMA can also be particularly difficult when the gastric tube lies in direct contact with the pedicle.

Although a left-sided approach is standard for minimally invasive cardiac surgery—CABG, including proximal anastomosis of the ascending aorta through left thoracotomy, this strategy was considered unsuitable in the present case because preoperative computed tomography indicated that safe exposure of the ascending aorta through a left thoracotomy would be technically challenging.

In our case, because the ascending aorta was positioned rightward in the mediastinum and the dilated gastric tube occupied the left side of the ascending aorta, a right minithoracotomy provided the safest and most direct access for proximal anastomosis. Conversely, the distal target coronary vessels were more favorably approached from the left thoracic cavity. For these reasons, bilateral minithoracotomies were selected, enabling safe exposure of both the ascending aorta and the target coronary arteries without disturbing the gastric conduit. This tailored strategy allowed complete 3-territory revascularization using saphenous vein grafts while avoiding extensive mediastinal dissection.

This experience demonstrates that minimally invasive cardiac surgery—CABG through bilateral minithoracotomy—can be a feasible and safe option for selected patients with previous esophagectomy and retrosternal gastric tube reconstruction. Detailed preoperative CT evaluation is essential for determining the optimal surgical strategy by defining the anatomic relationships among the gastric tube, the IMA, and ascending aorta.
